# Rapid and Efficient Microwave‐Assisted Friedländer Quinoline Synthesis

**DOI:** 10.1002/open.202000247

**Published:** 2020-11-05

**Authors:** Helen V. Bailey, Mary F. Mahon, Nigel Vicker, Barry V. L. Potter

**Affiliations:** ^1^ Medicinal Chemistry & Drug Discovery Department of Pharmacology University of Oxford Mansfield Road Oxford OX1 3QT UK; ^2^ Medicinal Chemistry Department of Pharmacy & Pharmacology University of Bath Claverton Down Bath BA2 7AY UK; ^3^ Department of Chemistry University of Bath Claverton Down Bath BA2 7AY UK

**Keywords:** Microwave, optimisation, quinoline, synthesis, X-ray crystallography

## Abstract

A microwave‐based methodology facilitates reaction of 2‐aminophenylketones with cyclic ketones to form a quinoline scaffold. Syntheses of amido‐ and amino‐linked 17β‐hydroxysteroid dehydrogenase type 3 inhibitors with a benzophenone‐linked motif were pursued using 2‐aminobenzophenone as building block. Two amido‐linked targets were achieved in modest yield, but when using microwave‐assisted reductive amination for the amino‐linked counterparts an unexpected product was observed. X‐ray crystallography revealed it as a quinoline derivative, leading to optimisation of a simple and efficient modification of Friedländer methodology. Using reagents and acetic acid catalyst in organic solvent the unassisted reaction proceeds only over several days and in very poor yield. However, by employing neat acetic acid as both solvent and acid catalyst with microwave irradiation at 160 °C quinoline synthesis is achieved in 5 minutes in excellent yield. This has advantages over the previously reported high temperatures or strong acids required, not least given the green credentials of acetic acid, and examples using diverse ketones illustrate applicability. Additionally, he unassisted reaction proceeds effectively at room temperature, albeit much more slowly.

## Graphical abstract

**Microwave‐assisted** reductive amination using 2‐aminobenzophenone and 1‐acetyl‐4‐piperidone as building blocks gave an unexpected quinolone‐based product, identified by single crystal X‐ray crystallography. With neat acetic acid as both solvent and acid catalyst and irradiation at 160 °C this quinoline synthesis was optimised as a rapid, simple and efficient green modification of the Friedländer methodology, applicable to a diverse set of ketones. This has advantages over the high temperatures or strong acids previously reported as required.

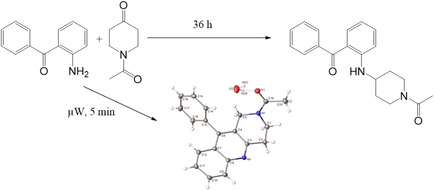

## 1 Introduction

Quinoline derivatives occur in numerous natural products and pharmaceutical entities, and especially in alkaloids. The archetypal example, quinine, was isolated in 1820 from the bark of the *Cinchona* tree and used in malaria treatment. Synthesis of the quinoline ring system is very important to the synthetic organic chemist and methodology has been widely discussed,[^1^, ^2^] with more recent work focusing considerably on green and clean methodologies.^[3]^ The structural core of quinolines can be made by many different methods, one of which is the Friedländer synthesis, originally published in 1882,^[4]^ a versatile and reliable reaction. It is traditionally a reaction in which an *o‐*aminobenzaldehyde is cyclised by reaction with an *α‐*methyleneketone in the presence of a base.^[5]^ The Friedländer synthesis can, however, be either acid‐ or base‐catalysed or it can even proceed without catalysis, although uncatalyzed reactions require very high temperatures, up to 220° C.^[1]^ In many cases it has been found that acid catalysis is more effective than base catalysis.^[6]^ Catalysts used for this reaction include hydrochloric acid,^[6]^ sulfamic acid,^[7]^ CuCl_2_,^[8]^ *p‐*toluenesulfonic acid,^[8]^ chlorotrimethylsilane^[10]^ and diphenylphosphate (DPP),^[11]^ amongst others.^[1]^ A comprehensive review on methodology was published in 2009.^[12]^

The pioneering work by Kappe *et al*. has been integral in the undersanding of the scope of microwave (μW)‐assisted reactions.^[13]^ Application of μW‐based technologies in organic synthesis now expedite large scale synthesis, batch processes, high‐throughput library synthesis and flow processes, with advantages in research and process chemistry and in drug discovery.[^13^, ^14^] In line with development of greener technologies for quinoline synthesis^[3]^ there have also been several μW‐enhanced procedures reported,[^6^, ^9^, ^10^, ^11^] demonstrating the advantages that such assisted synthesis can have upon this reaction, both in speeding it up and improving yields.^[15]^ However, novel and improved microwave procedures would be highly beneficial.

**Figure 1:**
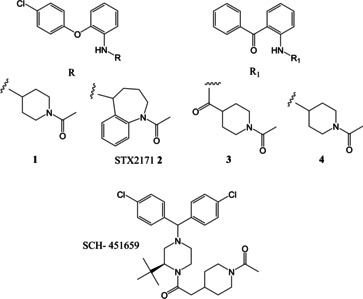
Diphenylether based inhibitors **1** and **2** and related initial targets in the benzophenone‐linked hydrophobic headgroup series: amido‐linked **3** and amino‐linked **4**. Structure of SCH‐451659 (STX1383).

In studies on the design of enzyme inhibitors as potential hormone‐dependent prostate cancer agents against the type 3 isozyme of enzyme 17β‐hydroxysteroid dehydrogenase (17β‐HSD3),[^16^, ^17^, ^18^] we designed a new diphenylether‐based series of leads that underwent early stage optimisation from an initial hit (**1** Figure 1) with an IC_50_ of 700 nM to the related **2** (STX2171), with potent and selective inhibitory activity and an IC_50_ of *ca* 200 nM, and that has been evaluated *in vivo*.^[19]^ Compound **2** lowered plasma testosterone levels and also inhibited androgen‐dependent tumour growth^[20]^ and provided the additional synthetic versatility of a different hydrophobic headgroup motif in comparison to the diphenylmethane motif of another potent *in vivo* active compound SCH‐451659 (STX1383)^[20]^ (Figure 1). To increase structural diversity further we also chose the related benzophenone‐based analogues as synthetic targets, with both associated amide and amine linkages, *eg* **3** and **4** respectively (Figure 1). Also desirable was exploitation, where possible, of our methodology for rapid μW‐assisted reductive amination of ketones with anilines, employing sodium triacetoxyborohydride as reducing agent, a procedure that improves both reaction rate and efficiency.^[21]^

**Scheme 1:**
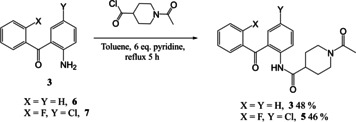
Synthesis of amide‐linked compounds **3** and **5** with a benzophenone‐linked headgroup.

**Scheme 2:**
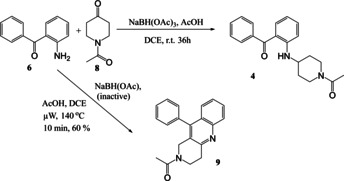
Synthesis of amine‐linked target **4** with a benzophenone headgroup and formation of the alternative product quinoline **9**.

Synthesis of the two initial amide‐linked targets **3** and **5** (Scheme 1) including a disubstituted benzophenone, was successful in moderate yield and did not require μW‐assistance. However, work on the related amine‐based target series, exemplified by the parent **4** and based upon reaction between 2‐aminophenyl ketone **6** and a cyclic ketone **8** (Scheme 2) utilised our μW‐assisted reductive amination strategy, but lead to an unexpected fused cyclic quinoline by‐product **9**, as confirmed by X‐ray crystallography. We now report here how this fortuitous observation was exploited and the reaction leading to it subsequently optimised to provide an attractive new variation on the μW‐assisted Friedländer quinoline synthesis that was then applied to a series of diverse ketones.

## 2 Results and Discussion

The straightforward synthesis of two initial amide‐linked targets in this series is shown in Scheme 1. Only one step was required from the 2‐aminobenzophenone starting material **6** to the final product **3** and both the unsubstituted **6** and halogen‐substituted 2‐amino‐5‐chloro‐2′‐fluorobenzophenone **7** were commercially available. However, a problem was encountered with reactivity in this series. An amide coupling reaction was attempted, with 1‐acetyl‐piperidine‐4‐carboxylic acid, using 1‐ethyl‐3‐(dimethylaminopropyl) carbodiimide as activating reagent, but this was unsuccessful. Next it was attempted to form the amide bond using a standard reaction with the corresponding acid chloride. However, the same reactivity problem was encountered and only starting materials were isolated. A literature study revealed two articles that used different, and more forcing, conditions to those attempted. Park *et al*.^[22]^ heated such starting materials in dichloromethane (DCM) with 6 eq. pyridine for 6 h and Kettlera *et al*.^[23]^ used a very similar method, heating the reaction mixture in toluene for 6 h. These two sets of conditions were adapted and the desired reaction was heated in toluene with 6 eq. pyridine for 6 h. This produced the correct products **3** and **5** in both cases with yields of 48 % and 46 % respectively.

The synthesis of one of the related amine‐linked targets **4** in this series from **6** and ketone **8** was carried out using a standard reductive amination method (Scheme 2) but, disappointingly however, the yield was low (36 %). It was hoped that this reaction could be speeded up and product yield improved and the reaction was repeated under conditions of the μW‐assisted reductive amination we developed earlier.^[21]^ However, this time a different major product **9** was obtained, in 60 % yield. It was found that an important difference between the two reactions was the supply of NaBH(OAc)_3_ used for the reductive amination. The reagent used in the second attempt was found to have degraded, and the alternative product had therefore been formed in the absence of active reducing agent.

**Figure 2:**
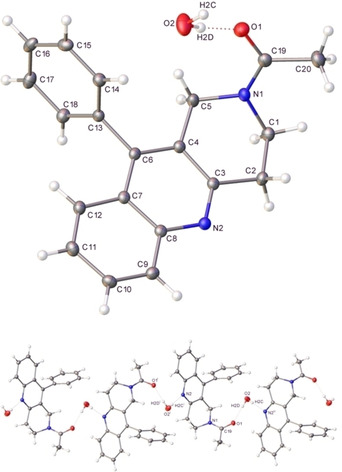
(a) Single crystal X‐ray structure of the Friedländer product **9** formed from 2‐aminobenzophenone **6** and 1‐acetyl‐piperidin‐4‐one **8**. Ellipsoids are represented at 30 % probability (b) Hydrogen‐bonding in the gross structure of **9** [symmetry operations: ^i^2 – *x*, – 1/2 +*y*, 1/2 – *z*, ^ii^2 – *x*, 1/2 +*y*, 1/2 – *z*] Figure 2 **(a**) shows the quinoline structure **9** identified while Figure 2 (b) shows a portion of its crystal packing structure. The included water molecule acts as a lattice ‘cement’ by hydrogen‐bonding to the quinoline nitrogen (N2) and the carbonyl oxygen (O1) of adjacent lattice molecules. Propogation of these hydrogen‐bonds by virtue of a screw axis parallel to *b* axis affords 1‐dimensional polymers in the gross array. [H2 C…N2^ii^, 2.073(4) Å ; O2‐H2C−N2^i^, 172(2)° ; H2D…O1, 1.958(3) Å ; O2‐H2D−O1, 174(3)° : ^ii^2 – *x*, 1/2 +*y*, 1/2 – *z*].

^1^H and ^13^C NMR and LCMS data were used to try to identify the unexpected and crystalline product, but were not conclusive. Single crystal X‐ray crystallography was therefore employed to determine the solid‐state structure of **9** (Figure 2) as the substituted quinoline 1‐(10‐Phenyl‐3,4‐dihydro‐1*H*‐benzo[*b*][1,6]‐naphthyridin‐2‐yl)‐ethanone (Scheme 2). The X‐ray structure of **9** shows one water molecule hydrogen bonding to the amide carbonyl in the quinoline and this is repeated in the unit cell. Thus, fortuitously an interesting modification of the Friedländer quinoline synthesis had been uncovered.

**Table 1:** Effects of time and temperature on the Friedländer synthesis of quinolines from **6** and **10**.

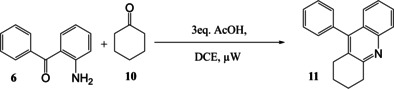			
Entry	Temperature (μW heating, °C)	Time (min)	Yield (%) ^[a]^
1	140	20	70
2	140	40	15
3	160	20	71
4	180	20	78
**5**	**200**	**20**	**86**

^[a]^ Isolated yield.

**Scheme 3:**
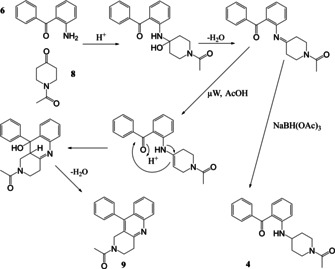
Mechanism for the formation of synthetic target **4** and Friedländer product **9** from a common Schiff base.

The Friedländer reaction is generally thought to proceed *via* the initial formation of a Schiff base, followed by an internal aldol condensation, although there is still some controversy about the mechanism.^[1]^ A mechanism for the formation of either **9** based upon this simplest interpretation or the originally targeted **4** via a common intermediate is shown in Scheme 3.

The reaction was therefore repeated as previously with μW‐assistance, except without inclusion of the NaBH(OAc)_3_ and the yield remained similar at *ca* 70 %. Literature reports of Friedländer syntheses generally show high yields, and often better than the 60–70 % yields exhibited so far under our conditions. 2‐Aminobenzophenones under thermal or basic conditions do not react with simple ketones such as cyclohexanone or beta‐keto esters,^[24]^ and acid catalysts such as sulfuric acid, hydrochloric acid, *p*‐TsOH,^[25]^ and polyphosphoric acids are more effective than basic catalysts for this reaction.[^26^, ^27^] This therefore chimed well with our preliminary observations and the use of acetic acid in an organic solvent. Many such known methods, however, require high temperatures, prolonged reaction times, and sometimes quite drastic reaction conditions and present work‐up difficulties, use stoichiometric or expensive reagents, and for this particular template give less than satisfactory yields due to side reactions.^[12]^ Development of more efficient and simple procedures for quinoline syntheses are needed and we reasoned that there should be considerable scope and value to attempt optimisation of our simple procedure. Investigations were therefore targeted at the discovery of optimal conditions for the reaction using our facile new μW procedure.

First, a model reaction between 2‐aminobenzophenone **6** and cyclohexanone **10** was used to optimise the conditions and a study into the effects of time and temperature was initiated. At room temperature alone the reaction proceeds incredibly slowly, with only an 18 % yield of product isolated after 3 days. The yield was dramatically increased by the use of μW assistance. The results are shown in Table 1. A solution of **6** (1 mmol), **10** (2 mmol) in DCE (2 mL) with AcOH (3 mmol) was heated in an μW vessel and increasing temperature leads to an increase in product yield of the quinoline 9‐Phenyl‐1,2,3,4‐tetrahydro‐acridine **11**. The highest yield was achieved at 200 °C, but above 200 °C there is no longer a significant increase observed and higher temperatures lead to degradation. Subsequently, in further optimisation of other parameters we showed there was no extra advantage at 200 °C and we eventually adopted 160 °C as the maximum practical temperature. It was also noted that the optimum reaction time is a maximum of 20 minutes, as reaction completion was not reached after 10 minutes and greater reaction times lead to reduced yields (Entry 2).

As with the original optimisation of our μW‐assisted reductive amination^[21]^ it was also crucial to investigate an array of solvents that possess different microwave properties (Table 2).[^14^, ^28^] It can be seen that the use of acetonitrile does not greatly affect product yield, compared to dichloroethane (DCE) (Entries 1 and 2), whereas use of toluene increases the yield from 78 % to 89 % (Entries 1 and 5).

**Table 2:** Effects of solvent on the Friedländer synthesis of quinolines from **6** and **10**.

Entry	Solvent	Temperature (μW heating, °C)	Time (min)	Yield (%) ^[a]^
1	DCE	180	20	78
2	acetonitrile	180	20	71
3	Toluene	140	20	78
4	Toluene	160	20	83
**5**	**Toluene**	**180**	**20**	**89**
6	Toluene	180	10	72

^[a]^ Isolated yield.

At this point, investigations were undertaken to attempt to drive the reaction near to completion, by assisting with the dehydration illustrated in Scheme 3. It was hoped that removal of water would help to encourage the forward equilibrium and therefore increase the yield of the reaction. Dehydrated magnesium sulphate (3 eq.) and 4 Å molecular sieves were tested, but reduced the isolated yield to 76 % and 54 % respectively.

Investigations were also made to see whether the acetic acid used so far could be successfully substituted for alternative reagents (Table 3). Results showed that, as expected, acid catalysis is required for protonation and dehydration, because without an acid present the yield is reduced to just 17 %, (Entry 1). Solid phase Amberlite ICR50 H‐form was tried but is also not a suitable alternative reagent, as this reduced the yield to just 7 %, (Entry 2). Conversely, it was found that, as reported in the literature, *p*‐toluenesulfonic acid (*p*‐TSA) is a very successful alternative reagent,^[9]^ and even when utilised in catalytic amounts it leads to excellent yields of 84 %, (Entries 3–5). However, the best condition identified from this study was the use of neat glacial acetic acid (AcOH) as the solvent, as operationally this is facile and excellent yields are obtained (Entry 6).

**Table 3:** Effects of acid catalyst and solvent on the μW‐assisted Friedländer synthesis of quinolines from **6** and **10**.

Entry	Solvent	Acid	Yield (%)^[a]^
1	Toluene	‐	17
2	Toluene	Amberlite ICR50 H‐form	7
3	Toluene	3eq. *p*‐TSA	86
4	Toluene	1eq. *p*‐TSA	84
5	Toluene	Cat. *p*‐TSA	84
**6**	–	**AcOH as solvent**	**87**

^[a]^ Isolated yield.

The use of AcOH both as solvent and acid catalyst is highly advantageous to this reaction in terms of results and operational simplicity. The new procedure was therefore simply to heat **6** (1 mmol) and **10** (2 mmol) in AcOH (2 mL) in a μW tube. This lead to higher yields and shorter reaction times (Table 4). The best yields though are obtained with just a 5 or 10 minute heating period at 160 °C (Entries 2 and 3), with almost optimal yields obtained. Longer heating periods lead to a small decrease in isolated yield (Entry 4).

**Table 4:** Effects of time and temperature on the Friedländer quinoline synthesis from **6** and **10** when AcOH is used as the solvent.

Entry	Temperature (μW heating, °C)	Time (min)	Yield (%)^[a]^
1	100	5	79
**2**	**160**	**5**	**94**
3	160	10	94
4	160	20	87

^[a]^ Isolated yield.

Following the identification of the optimal conditions of AcOH as solvent and heating in a μW vessel for just 5 minutes at 160 °C, further investigations were undertaken to examine more general applicability. A larger scale reaction was carried out using 500 mg of 2‐aminobenzophenone **6** (2.5 mmol) in the same volume of AcOH (2 mL), thus more than doubling the concentration of starting materials. A yield of 95 % was obtained and thus there was no difference due to scale, showing the process to have high potential for larger scale reactions, where minimal solvent use is desired.

Thus, with a short reaction time, a cheap solvent and little excess reagent the attractions of this new procedure are evident. Interestingly, this system is actually now so efficient that the reaction proceeds efficiently even at room temperature (89 % yield after 24 h), potentially very useful should μW technology not be available. While clearly the use of AcOH is still not perfect in a “green” sense, if reaction rapidity is not an issue its use as both a solvent and catalyst could certainly be seen to be a greener alternative to that of μW technology, organic solvents, mineral acids and more complex catalysts etc in line with current trends.^[3]^ Indeed, when solvents are evaluated according to low environmental risk acetic acid is commonly recognized as green. In a recent study of 78 common solvents so classified, acetic acid ranked as 6^th^ best.^[29]^

Investigations were also carried out to explore the effect, if any, of reducing the relative amount of ketone used (Table 5). There was little difference in yields observed when using 1, 1.5 or 2 equivalents of cyclohexanone **10**. For this reason, the amount of ketone used in all subsequent reactions was reduced to 1.5 equivalents. An excess was used to ensure complete consumption of the amine. This ratio could be further reduced if required by a particular application.

**Table 5:** Use of differing amounts of ketone on Friedländer quinoline synthesis from **6** and **10**.

Entry	Equivalents of cyclohexanone used	Yield (%)^[a]^
1	2	94
**2**	**1.5**	**92**
3	1	87

^[a]^ Isolated yield.

Now that conditions had substantially been optimised, this new procedure was applied to a small range of different starting ketones and an aldehyde (Table 6). Excellent yields of quinolines **11**–**16** were obtained in all cases. The reaction with 2‐hexanone showed some degree of regioselectivity as the two possible products **14 a** and **14 b** were obtained in a 1: 1.9 ratio respectively (Entry 4). In one other case (Entry 6) *o*‐amino‐acetophenone **17** ^[4]^ was substituted for **6**, giving an excellent yield of quinoline **16** (91 %).

**Table 6:** Microwave‐assisted Friedländer synthesis of diverse quinolines (using 1.5 equivalents of ketone).

Entry	Starting Material	Ketone	Product	Yield (%)^[a]^
1	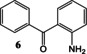	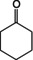	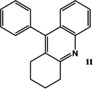	94
2	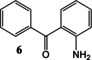	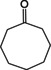	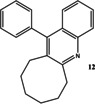	93
3	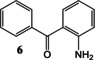	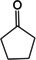	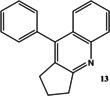	99
4	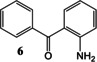	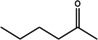	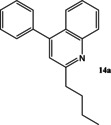	23^b^
			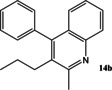	43^b^
5	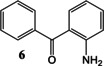	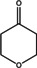	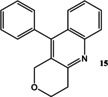	99
6	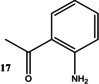	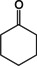	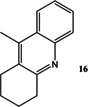	91

^[a]^ Isolated yield. ^[b]^ Regioisomers obtained, 1 : 1.9, overall yield 66 %.

**Scheme 4:**
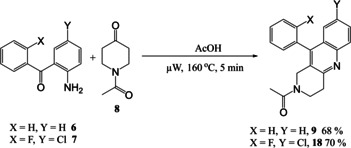
Synthesis of two quinoline‐based products using optimised μW methodology.

Application of our new procedure was applied to the benzophenones in Scheme 1, affording the expected compounds in excellent yields of 68 % and 70 % respectively (Scheme 4). However, when 1‐acetyl‐4‐piperidone **8** is used as the ketone, there is an additional problem concerning the removal of excess starting ketone. The product and the ketone co‐elute so cannot be separated by flash chromatography. Although this problem had been reduced by using only 1.5 equivalents of ketone, it was still significant. Luckily, it was easily solved by stirring the crude product with the electrophile scavenger resin PS−TsNHNH_2_ ^[30]^ in DCM for 1 hour, the resin simply removed by filtration and product purified by flash chromatography. This extra purification may be part of the reason why these yields are lower than with other compounds (Table 6) because the overall yield for this step did not actually show any improvement following the optimisation process. The new conditions are, however, faster and operationally simpler.

**Scheme 5:**
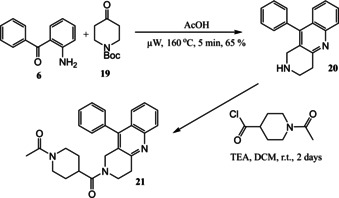
Synthesis of an extended quinoline‐based analogue using optimised μW methodology.

Given the results for biological activity from the parent diphenyl ether series[^19^, ^20^] it was likely that these quinoline based compounds (**9** and **18**) might be too short for optimal 17β‐HSD3 enzyme inhibitory activity, so an extended analogue, more akin to SCH‐451659 (STX1383) **21** was targeted (Scheme 5). The initial step between 2‐aminobenzophenone **6** and the suitably protected 1‐Boc‐4‐piperidone **19** did not, however, proceed as envisaged and did not produce the corresponding Boc‐protected intermediate. The reaction lead instead to the deprotected free amine intermediate **20**. Although not as planned, this made the synthetic route one step shorter, as the Friedländer synthesis and deprotection occured in one step and in a good yield of 65 %. This does, however, show that some acid labile groups are not tolerated by our new Friedländer methodology and other amine protecting groups may thus prove more suitable. The free amine **20** was then reacted with 1‐acetyl‐piperidine‐4‐carbonyl chloride using standard conditions to give **21**. Amine **20** is an attractive advanced intermediate in its own right for library construction using parallel synthesis in lead generation/optimisation.

## 3 Conclusions

Compounds possessing a quinoline scaffold exhibit a wide spectrum of pharmacological activities and new methodology for their synthesis is highly pertinent. In summary, we have demonstrated here that the quinoline scaffold can be efficiently and rapidly constructed in excellent yield in only *ca* 5 min *via* the microwave‐assisted reaction of a 2‐aminophenyl ketone with cyclic or acyclic ketones using neat acetic acid both as solvent and catalyst. Using dilute acetic acid in organic solvent and without microwave assistance the reaction proceeds only very slowly and in very poor yield. Optimisation of temperature, solvent, acid catalyst, time and reagent ratio was studied and a focused range of diverse quinolines was produced to illustrate reaction versatility. Moreover, should microwave technology not be readily available the reaction proceeds cleanly and in high yield alone even at room temperature, albeit over a much longer timeframe. While highly efficient, the operational simplicity of this methodology is also greener than a number of more traditional synthetic approaches. This methodology should add to the armoury of techniques established for Friedländer quinoline synthesis[^2^, ^3^, ^12^, ^31^, ^32^, ^33^] and find diverse applications in organic synthesis and for medicinal chemistry.

### Experimental SectionMaterials and Methods

All chemicals were purchased from Aldrich Chemical Co. (Gillingham, UK) or Lancaster Synthesis (Morecambe, U.K.). All organic solvents were supplied by Fisher Scientific (Loughborough, U.K.). Reactions using anhydrous solvents were carried out under nitrogen. Thin layer chromatography (TLC) was performed on precoated plates (Merck TLC aluminium sheets silica gel 60 F_254_). Product(s) and starting material(s) were detected by either viewing under UV light and/or treating with a suitable staining system, for example vanillin, followed by heating. Flash column chromatography was performed on silica gel (Sorbsil/Matrex C60) or using Argonaut prepacked columns with a Flashmaster^TM^. IR spectra were recorded in DCM solutionin Perkin‐Elmer Spectrum RXI FT‐IR spectrometer cells and peak positions are expressed in cm^−1^. ^1^H NMR (270 MHz or 400 MHz) and *DEPT*‐edited ^13^C NMR (68 MHz or 101 MHz) spectra were recorded with a Jeol Delta 270 or a Varian Mercury VX 400 NMR spectrometer and chemical shifts are reported in parts per million (ppm). HPLC analyses were performed on a Waters Millenium 32 instrument equipped with a Waters 996 PDA detector. A Waters Radialpack C18 reversed phase column (8×100 mm) was eluted with the solvent system specified at 1 mL/min. Microwave irradiation was carried out using a CEM Discover® instrument (CEM Microwave Discovery Ltd, Buckingham, UK). FAB low and high resolution mass spectra were recorded at the Mass Spectrometry Service Centre, University of Bath, using *m*‐nitrobenzyl alcohol (NBA) as the matrix. ES and APCI low resolution mass spectra were obtained on a Waters Micromass ZQ. Elemental analyses were performed by the Microanalysis Service, University of Bath. Melting points were determined using a Reichert‐Jung Thermo Galen Kofler block and are uncorrected.

### X‐Ray Crystallography

Data for **9** ^[34]^ were obtained at 150 K using a Nonius Kappa CCD diffractometer and Mo−Kα radiation. Solution and refinement of the model were effected using the SHELX[^35^, ^36^] suite of programs *via* Olex‐2.^[37]^ Refinement was unremarkable and the only point of note is that the hydrogen atoms, in the included water molecule, were readily located and refined at a distance of 0.89 Å from O2. For full details see the Supplementary Information, Table S1.

Crystallographic data for **9** have been deposited with the Cambridge Crystallographic Data Centre as supplementary publication CCDC 2007130. Copies of these data can be obtained free of charge on application to CCDC, 12 Union Road, Cambridge CB2 1EZ, UK [fax(+44) 1223 336033, e‐mail: deposit@ccdc.cam.ac.uk

### 1‐Acetyl‐piperidine‐4‐carboxylic acid (2‐benzoyl‐phenyl)‐amide 3

The above compound was synthesised adapting methodology reported by Kettlera *et al*.^[23]^ 1‐Acetylpiperidine‐4‐carbonyl chloride (173 mg, 0.91 mmol) was dissolved in toluene (5 mL) and this was added to a solution of 2‐aminobenzophenone (150 mg, 0.76 mmol) and pyridine (0.38 mL, 4.6 mmol) in toluene (15 mL). The resulting mixture was heated at reflux for 6 h. The toluene was removed *in‐vacuo* and aqueous HCl solution (1 M) was added. The mixture was then extracted with DCM, dried (MgSO_4_), filtered and solution evaporated to dryness. The crude product was purified by flash chromatography (0–10 % MeOH in DCM), to yield the desired product as a white solid (129 mg, 48 %). R_f_ 0.44 (10 % MeOH in EtOAc), mp: 191–193 °C, LCMS: *t*_r_=0.94 min (95 % MeOH in water), *m/z* M−H 349.14, HPLC: *t*_r_=1.68 min (90 % acetonitrile in water), >99 %, ^1^H NMR (CDCl_3_, 270 MHz,): δ 1.67–1.87 (2H, m, CH_2_), 2.01–2.09 (2H, m, CH_2_), 2.09 (3H, s, CH_3_), 2.50–2.61 (1H, m, CH), 2.60–2.75 (1H, m, 1/2 CH_2_), 3.08–3.17 (1H, m, 1/2 CH_2_), 3.89 (1H, d, *J*=13.4 Hz, 1/2 CH_2_), 4.64 (1H, d, *J*=13.4 Hz, 1/2 CH_2_), 7.09 (1H, td, *J*=0.97, 7.4 Hz, ArH), 7.46–7.51 (2H, m, ArH), 7.55–7.63 (3H, m, ArH), 7.67–7.69 (2H, m, ArH), 8.63–8.66 (1H, m, ArH), 11.10 (1H, s, NH). ^13^C NMR (CDCl_3_, 68 MHz): 21.6 (CH_3_), 28.5, 28.9, 41.1 (CH_2_), 44.7 (CH), 45.9 (CH_2_), 121.5, 122.4 (ArCH), 123.1 (ArC), 128.5, 129.9, 132.7, 134.0, 134.7 (ArCH), 138.7, 140.7 (ArC), 167.5, 169.0, 173.2 (CO); HRMS: Calcd for C_21_H_22_N_2_O_3_ (M+Na)^+^373.1526, found (M+Na)^+^373.1523; Anal. calcd for C_21_H_22_N_2_O_3_: C 72.00, H 6.29, N 8.00 %, found: C 71.6, H 6.29, N 8.00 %.

### 1‐Acetyl‐piperidine‐4‐carboxylic acid [4‐chloro‐2‐(2‐fluoro‐benzoyl)‐phenyl]‐amide 5

The above compound was synthesised adapting methodology reported by Kettlera *et al*.^[23]^ 1‐Acetylpiperidine‐4‐carbonyl chloride (136 mg, 0.72 mmol) was dissolved in toluene (5 mL) and this was added to a solution of 2‐amino‐5‐chloro‐2′‐fluorobenzophenone (150 mg, 0.6 mmol) in toluene (15 mL). The reaction was heated at reflux for 2 h. The toluene was removed *in vacuo* and 1 M HCl was added, the crude residue was then extracted with DCM, dried (MgSO_4_), solution filtered and evaporated to dryness. The crude product was purified by flash chromatography (0–10 % MeOH in DCM) to yield the desired product as an off‐white solid, (112 mg, 46 %). R_f_ 0.35 (EtOAc), mp: 202–204 °C, LCMS: *t*_r_=1.07 min (95 % MeOH in water), *m/z* M−H 401.10, HPLC: *t*_r_=1.79 min (90 % acetonitrile in water), >99 %, ^1^H NMR (CDCl_3_, 270 MHz,): δ 1.65–1.90 (2H, m, CH_2_), 2.02–2.08 (5H, m, CH_2_ and CH_3_), 2.51–2.75 (2H, m, 1/2 CH_2_ and CH), 3.08–3.18 (1H, m, 1/2 CH_2_), 3.89 (1H, d, *J*=13.3 Hz, 1/2 CH_2_), 4.63 (1H, d, *J*=13.3 Hz, 1/2 CH_2_), 7.15–7.31 (2H, m, ArH), 7.41–7.61 (4H, m, ArH), 8.71 (1H, d, *J*=8.9 Hz, ArH), 11.35 (1H, br.s, NH). ^13^C NMR (CDCl_3_, 68 MHz): 21.5 (CH_3_), 28.5, 28.9, 41.0 (CH_2_), 44.7 (CH), 45.8 (CH_2_), 116.6, 122.5 (ArCH), 123.7 (ArC), 130.3, 133.4, 133.8, 133.9, 135.4 (ArCH), 139.7, 157.7, 161.5 (ArC), 169.0, 173.3, 196.3 (CO); HRMS: Calcd for C_21_H_20_ClFN_2_O_3_ (M+H)^+^403.1219, found (M+H)^+^403.1218; Anal. calcd for C_21_H_20_ClFN_2_O_3_: C 62.61, H 5.00, N 6.95 %, found: C 62.78, H 5.01, N 6.87 %.

### 1‐[4‐(2‐Benzoyl‐phenylamino)‐piperidin‐1‐yl]‐ethanone 4

To a solution of 2‐aminobenzophenone (197 mg, 1 mmol) and 1‐acetyl‐4‐piperidone (282 mg, 2 mmol) in DCE (3 mL) was added NaBH(OAc)_3_ (530 mg, 2.5 mmol) and AcOH (0.18 mL). The resulting solution was stirred at r.t. for 36 h. NaHCO_3_ was added and the mixture was extracted with DCM. The crude product was purified by flash chromatography (0–10 % MeOH in EtOAc) to yield the desired product as a yellow oil (116 mg, 36 %). R_f_ 0.58 (10 % MeOH in EtOAc), LCMS: *t*_r_=4.39 min (50 % to 95 % MeOH in water), *m/z* (M+H)^+^323.14, HPLC: *t*_r_=2.03 min (90 % acetonitrile in water), 95 %, ^1^H NMR (CDCl_3_, 270 MHz,): δ 1.48–1.68 (2H, m, CH_2_), 2.00–2.10 (5H, m, CH_3_ and CH_2_), 3.10–3.34 (2H, m, CH_2_), 3.67–3.82 (2H, m, CH_2_), 4.19–4.28 (1H, m, CH_2_), 6.50–6.56 (1H, m, ArH), 6.78 (1H, d, *J*=8.4 Hz, ArH), 7.33–7.59 (7H, m, 6ArH and NH), 8.78 (1H, d, *J*=7.2 Hz, ArH). ^13^C NMR (CDCl_3_, 68 MHz): 21.6 (CH_3_), 31.4, 32.2, 39.8, 44.7 (CH_2_), 48.4 (CH), 111.8, 114.1, 128.2, 129.1, 130.9, 135.1, 136.0 (ArCH), 150.6, 169.0 (CO). HRMS: Calcd for C_20_H_22_N_2_O_2_ (M+H+Na)^+^345.1573, found (M+H+Na)^+^345.1565.

### General Procedure for the Friedländer Cyclisation

The desired benzophenone (1 mmol) and ketone (2 mmol) were dissolved in AcOH (2 mL). The resulting solution was subjected to microwave heating for 5 min at 160 °C. Saturated NaHCO_3_ was then added and the mixture was extracted with DCM, dried (MgSO_4_), filtered, the solution concentrated *in vacuo* and the residue purified by flash chromatography to yield the desired quinoline product as detailed below.

### 1‐(10‐Phenyl‐3,4‐dihydro‐1*H*‐benzo[*b*][1,6]naphthyridin‐2‐yl)‐ethanone 9

Following the general procedure for the Friedländer Cyclisation and flash chromatography it was found that the product was still contaminated with the 1‐acetyl‐4‐piperidone starting material. This was removed by use of PS−TsNHNH_2_. The crude material was dissolved in DCM (∼10 mL/g) and the resin was added (3 eq, 2.8 mmol/g), this was then stirred at r.t. for 1 h. The resin was removed by filtration and flash chromatography isolated the desired product as a white solid (206 mg, 68 %): R_f_ 0.33 (10 % MeOH in DCM); mp: 163–165 °C (from hexane), (lit. 166–167 °C^[38]^); LCMS: *t*_r_=0.95 min (95 % MeOH in water); HPLC: *t*_r_=1.83 min (90 % acetonitrile in water), 99 %; ^1^H NMR (CDCl_3_, 270 MHz,): δ 1.95, 2.16 (3H, CH_3_), 3.29–3.32 (2H, m, CH_2_), 3.83–3.99 (2H, m, CH_2_), 4.43, 4.60 (2H, CH_2_), 7.23–7.28 (2H, m, ArH), 7.38–7.39 (2H, m, ArH), 7.47–7.59 (3H, m, ArH), 7.62–7.70 (1H, m, ArH), 8.04 (1H, t, *J*=6.6 Hz, ArH); ^13^C NMR (CDCl_3_, 101 MHz): 21.4, 21.9 (CH3), 32.8, 33.9, 39.8, 42.6, 43.8, 46.5 (CH2), 124.0, 124.6 (ArC), 125.9, 126.0, 126.2, 126.3 (ArCH), 126.4, 126.8 (ArC), 128.4, 128.5, 128.6, 128.7, 128.9, 129.0, 129.1, 129.3, 129.5 (ArCH), 135.1, 135.2, 145.1, 146.1, 146.6, 146.9, 155.3, 156.3 (ArC), 169.3, 169.4 (CO); IR: 2800 (m), 1650 (s), 1300 (m), 1030 (m); *m/z* M+H 303.09; HRMS: Calcd for C_20_H_18_N_2O_ (M+H)+303.1492, found (M+H)+303.1491; Anal. calcd for C_20_H_18_N_2_O (+ 1/2 mole AcOH): C 75.9, H 6.1, N 8.4 %, found: C 76.1, H 6.2, N 9.0 %. This compound was previously synthesised *via* a different method by Khaldeeva *et al*.^[38]^

### 9‐Phenyl‐1,2,3,4‐tetrahydro‐acridine 11

Following the general procedure for the Friedländer Cyclisation the desired product was isolated (246 mg, 94 %): R_f_ 0.7 (10 % MeOH in DCM), mp: 137–139 °C, (lit. 139–141 °C^[39]^); ^1^H NMR (CDCl_3_, 270 MHz,): δ 1.7–1.79 (2H, m, CH_2_), 1.90–2.01 (2H, m, CH_2_), 2.59 (2H, t, *J*=6.5 Hz, CH_2_), 3.19 (2H, *J*=6.4 Hz, CH_2_), 7.20–7.31 (4H, m, ArH), 7.4–7.65 (4H, m, ArH), 8.00 (1H, d, *J*=8.2 Hz, ArH); ^13^C NMR (CDCl_3_, 68 MHz): 23.0, 23.1, 28.2, 34.4 (CH_2_), 125.5, 125.9 (ArCH), 127.0 (ArC), 127.8, 128.4, 128.7, 129.2 (ArCH), 137.2, 147.0, 159.2 (ArC); *m/z* M+H 400.50; LCMS: *t*_r_=4.19 min (50 % to 95 % MeOH in water at 0.5 mL/min to 1.0 mL/min over 5 min). This compound has been previously synthesised using a different method, by Shaabani *et al*.^[39]^

### 12‐Phenyl‐6,7,8,9,10,11‐hexahydro‐cycloocta[*b*]quinoline 12

Following the general procedure for the Friedländer Cyclisation the desired product was isolated as an off‐white solid (267 mg, 93 %): R_f_ 0.3 (DCM); mp: 120–122 °C; HPLC: *t*_r_=4.9 min (90 % acetonitrile in water), 98 %, ^1^H NMR (CDCl_3_, 270 MHz,): δ 1.30–1.51 (6H, m, CH_2_), 1.90–1.98 (2H, m, CH_2_), 2.76 (2H, t, *J*=5.7 Hz, CH_2_), 3.22 (3H, t, *J*=6.2 Hz, CH_2_), 7.18–7.32 (4H, m, ArH), 7.45–7.51 (3H, m, ArH), 7.55–7.52 (1H, m, ArH), 8.05 (1H, d, *J*=7.9 Hz, ArH); ^13^C NMR (CDCl_3_, 68 MHz): 25.9, 26.8, 28.2, 31.2, 31.4, 36.5 (CH_2_), 125.5, 126.2 (ArCH), 127.3 (ArC), 127.7, 128.3, 128.4, 128.6, 129.4 (ArCH), 131.9, 137.7, 146.5, 163.6 (ArC); LCMS: *t*_r_=1.78 min (95 % MeOH in water), *m/z* M+H 288.10; HRMS: Calcd for C_21_H_21_N (M+H)^+^288.1747, found (M+H)^+^288.1756; Anal. calcd for C_21_H_21_N: C 87.76, H 7.36, N 4.87 %, found: C 87.40, H 7.32, N 4.87 %. This compound has been previously synthesised using a different method, by Bose *et al*.^[40]^

### 9‐Phenyl‐2,3‐dihydro‐1*H*‐cyclopenta[*b*]quinoline 13

Following the general procedure for the Friedländer Cyclisation the desired product was isolated as an off‐white solid (243 mg, 99 %): R_f_ 0.32 (DCM); mp: 132–134 °C, (lit. 131–132 °C^[39]^); HPLC: *t*_r_=3.2 min (90 % acetonitrile in water), >99 %; ^1^H NMR (CDCl_3_, 270 MHz): δ 2.10–2.20 (2H, m, CH_2_), 2.89 (2H, t, *J*=7.4 Hz, CH_2_), 3.23 (2H, t, *J*=7.7 Hz, CH_2_), 7.32–7.65 (8H, m, ArH), 8.06–8.08 (1H, m, ArH); ^13^C NMR (CDCl_3_, 68 MHz): 23.6, 30.4, 35.2 (CH_2_), 126.6, 125.7 (ArCH), 126.3 (ArC), 128.1, 128.4, 128.6, 128.7, 129.4 (ArCH), 133.8, 136.8, 143.0, 147.9, 167.5 (ArC); LCMS: *t*_r_=1.5 min (95 % MeOH in water), *m/z* M+H 245.90; HRMS: Calcd for C_18_H_15_N (M+H)^+^246.1277, found (M+H)^+^246.1250. Anal. calcd for C_18_H_15_N: C 88.13, H 6.16, N 5.71 %, found: C 87.90, H 6.13, N 5.76 %. This compound has been previously synthesised using a different method, by Shaabani *et al*.^[39]^

### 2‐Butyl‐4‐phenyl‐quinoline 14 a and 2‐methyl‐4‐phenyl‐3‐propyl‐quinoline 14 b

Following the general procedure for the Friedländer Cyclisation the desired products were synthesised and isolated. Overall yield (171 mg, 66 %), selectivity 1 : 1.9 (A : B).

**14 a: 2‐Butyl‐4‐phenyl‐quinoline** was isolated as an off‐white oil (59 mg, 23 %): R_f_ 0.38 (DCM); HPLC: *t*_r_=4.75 min (90 % acetonitrile in water), 93 %; ^1^H NMR (CDCl_3_, 270 MHz,): 0.96 (3H, t, *J*=7.2 Hz, CH_3_), 1.39–1.52 (2H, m, CH_2_), 1.79–1.86 (2H, m, CH_2_), 2.99 (2H, t, *J*=7.9 Hz, CH_2_), 7.39–7.59 (7H, m, ArH), 7.62–7.71 (1H, m, ArH), 8.08–8.11 (1H, m, ArH); ^13^C NMR (CDCl_3_, 68 MHz): 15.0 (CH_3_), 22.9, 32.4, 39.3 (CH_2_), 121.7 (ArCH), 125.3 (ArC), 125.7, 125.8, 128.4, 128.6, 129.3, 129.3, 129.6 (ArCH), 138.4, 148.5, 148.6, 148.6, 167.8 (ArC); LCMS: *t*_r_=1.54 min (95 % MeOH in water), *m/z* M+H 261.96; HRMS: Calcd for C_19_H_19_N (M+H)^+^262.1590, found (M+H)^+^262.1598. This compound has been previously synthesised *via* a different route by Kobayashi *et al*.^[41]^

**14 b**: **2‐Methyl‐4‐phenyl‐3‐propyl‐quinoline** was isolated as a white solid (112 mg, 43 %): R_f_ 0.24 (DCM), mp: 114–116 °C; HPLC: *t*_r_=3.87 min (90 % acetonitrile in water), >99 %; ^1^H NMR (CDCl_3_, 270 MHz,): δ 0.81 (3H, t, *J*=7.4 Hz, CH_3_CH_2_), 1.40–1.49 (2H, m, CH_2_), 2.48–2.54 (2H, m, CH_2_), 2.80 (3H, s, CH_3_Ar), 7.20–7.32 (3H, m, ArH), 7.44–7.51 (4H, m, ArH), 7.54–7.61 (1H, m, ArH), 8.03 (1H, dd, *J*=0.5, 8.4 Hz, ArH); ^13^C NMR (CDCl_3_, 68 MHz): 14.5 (CH_3_), 23.6 (CH_2_), 23.9 (CH_3_), 32.5 (CH_3_), 125.4, 126.2 (ArCH), 127.1 (ArC), 127.6, 128.2, 128.3, 128.4, 129.3 (ArCH), 132.1, 137.4, 145.9, 146.5, 158.6 (ArC); LCMS: *t*_r_=1.41 min (95 % MeOH in water), *m/z* M+H 261.96; HRMS: Calcd for C_19_H_19_N (M+H)^+^262.1590, found (M+H)^+^262.1591; Anal. calcd for C_19_H_19_N: C 87.31, H 7.33, N 5.36 %, found: C 86.9, H 7.48, N 5.26 %.

### 10‐Phenyl‐3,4‐dihydro‐1H‐pyrano[4,3‐*b*]quinoline 15

Following the general procedure for the Friedländer Cyclisation the desired product was isolated as a yellow solid (260 mg, 99 %): R_f_ 0.65 (10 % MeOH in DCM); mp: 146–148 °C (lit. 130 °C^[42]^); HPLC: *t*_r_=2.41 min (90 % acetonitrile in water), >99 %; ^1^H NMR (CDCl_3_, 270 MHz,): δ 1.93 (2H, s, CH_2_), 2.24 (2H, s, CH_2_), 4.80 (2H, s, CH_2_), 7.02–7.12 (2H, m, ArH), 7.21–7.30 (4H, m, ArH), 8.20 (1H, dd, *J*=7.7 Hz, ArH); ^13^C NMR (CDCl_3_, 68 MHz): 22.1, 24.6 (CH_2_), 49.8 (CH_2_), 118.1, 120.7, 122.1, 123.0, 124.0 (ArCH), 125.0 (ArC), 129.2 (ArCH), 129.8 (ArC), 130.1, 130.2, 131.5 (ArCH), 137.7, 153.2, 153.8 (ArC), 169.5, 172.7 (CO); LCMS: *t*_r_=1.35 min (95 % MeOH in water), *m/z* M−H 262.09; HRMS: Calcd for C_18_H_15_NO _3_ (M+H)^+^262.1226, found (M+H)^+^262.1223. Anal. calcd for C_18_H_15_NO: C 82.73, H 5.79, N 5.36 %, found: C 82.30, H 5.74, N 5.38 %. This compound was previously synthesised, using a different route, by Kempter *et al*.^[42]^

### 9‐Methyl‐acridine 16

Following the general procedure for the Friedländer Cyclisation the desired product was isolated as a yellow oil (180 mg, 91 %): R_f_ 0.14 (DCM), LCMS: *t*_r_=1.32 min (95 % MeOH in water); HPLC: *t*_r_=4.8 min (90 % acetonitrile in water), >99 %; ^1^H NMR (CDCl_3_, 270 MHz,): δ 1.88–1.92 (4H, m, 2CH_2_), 2.52 (3H, s, CH_3_), 2.86 (2H, br.s, CH_2_), 3.10–3.12 (2H, m, CH_2_), 7.41–7.47 (1H, m, ArH), 7.59 (1H, td, *J*=1.4, 6.9 Hz, ArH), 7.93 (1H, dd, *J*=0.81, 8.5 Hz, ArH), 8.00 (1H, d, *J*=8.5 Hz, ArH), ^13^C NMR (CDCl_3_, 68 MHz): 13.8 (CH_3_), 22.7, 23.2, 27.2, 33.9 (CH_2_), 123.4, 125.5 (ArCH), 126.9 (ArC), 128.3, 128.5 (ArCH), 128.6, 128.9, 142.1, 145.3, 158.5, 171.0 (ArC); *m/z* M+H 197.71; HRMS: Calcd for C_14_H_11_N (M+H)^+^198.1277, found (M+H)^+^198.1271. This compound was previously synthesised, using a different route, by Wang *et al*.^[43]^

### 1‐[8‐Chloro‐10‐(2‐fluoro‐phenyl)‐3,4‐dihydro‐1*H*‐benzo[*b*][1,6] naphthyridin‐2‐yl] ethanone 19

Following the general procedure for the Friedländer Cyclisation and flash chromatography it was found that the product was still contaminated with 1‐acetyl‐4‐piperidone. This was removed by use of PS−TsNHNH_2_, the crude material was dissolved in DCM (∼10 mL/g) and the resin was added (3eq, 2.8 mmol/g), this was stirred at r.t. for 1 h. The resin was removed by filtration and flash chromatography isolated the desired product as a white solid (248 mg, 70 %): R_f_ 0.30 (EtOAc); mp: 196–199 °C; HPLC: *t*_r_=2.07 min (90 % acetonitrile in water), 98 %; ^1^H NMR (CDCl_3_, 270 MHz,): δ 2.18 (3H, s, CH_3_), 3.27–3.33 (2H, m, CH_2_), 3.81–4.00 (2H, m, CH_2_), 4.37–4.45 (1H, m, 1/2 CH_2_), 4.75 ‐ 4.82 (1H, m, 1/2 CH_2_), 7.19–7.39 (4H, m, ArH), 7.50–7.64 (2H, m, ArH), 7.96–8.02 (1H, m, ArH); ^13^C NMR (CDCl_3_, 101 MHz): 21.7, 21.8 (CH_3_), 32.9, 34.0, 39.7, 42.4, 43.7, 46.4 (CH_2_), 116.7 (d, *J*=21.8 Hz, ArCH), 122.0 (ArC), 124.3 (ArCH), 125.1 (d, *J*=3.7 Hz, ArCH), 126.8 (ArC), 130.5, 130.6, 131.0, 131.5 (ArCH), 132.5, 139.0, 155.7, 157.6, 161.3 (ArC), 169.2, 169.4 (CO); LCMS: *t*_r_=1.03 min (95 % MeOH in water), *m/z* M+H 355.19; HRMS: Calcd for C_20_H_16_ClFN_2_O (M+Na)^+^377.0817, found (M+Na)^+^377.0827; Anal. calcd for C_20_H_16_ClFN_2_O: C 67.70, H 4.55, N 7.90 %, found: C 67.8, H 4.57, N 7.84 %.

### 10‐Phenyl‐1,2,3,4‐tetrahydro‐benzo[*b*][1,6]naphthyridine 20

Following the general procedure for the Friedländer Cyclisation using 2‐aminobenzophenone (197 mg) and 1‐Boc‐4‐piperidone (300 mg) and subsequent purification (0–10 % MeOH in EtOAc, with 5 % TEA) the desired product was isolated as a yellow oil (170 mg, 65 %): R_f_ 0.12 (10 % MeOH in EtOAc); HPLC: *t*_r_=3.20 min (90 % acetonitrile in water), 96 %; ^1^H NMR (CDCl_3_, 270 MHz,): δ 2.05 (1H, br.s, NH), 3.21–3.30 (4H, m, 2CH_2_), 3.84 (2H, s, CH_2_), 7.20–7.25 (3H, m, ArH), 7.31–7.33 (2H, m, ArH), 7.45–7.53 (2H, m, ArH), 7.57–7.63 (1H, m, ArH), 8.01 (1H, dd, *J*=0.8, 9.1 Hz, ArH); ^13^C NMR (CDCl_3_, 68 MHz): 34.3, 44.1, 47.5 (CH_2_), 125.8, 125.9 (ArCH), 126.6, 127.0 (ArC), 128.2, 128.5, 128.8, 129.0 (ArCH), 136.0, 144.9, 146.8, 156.6 (ArC); LCMS: *t*_r_=1.70 min (95 % MeOH in water), *m/z* M+H 261.08.

### 1‐[4‐(10‐Phenyl‐3,4‐dihydro‐1*H*‐benzo[*b*][1,6]naphthyridine‐2‐carbonyl)‐cyclohexyl]‐ethanone 21

A solution of 10‐phenyl‐1,2,3,4‐tetrahydro‐benzo[*b*][1,6]naphthyridine (195 mg, 0.38 mmol) in DCM (10 mL) was cooled in an ice bath and to this was added 1‐acetyl‐piperidine‐4‐carbonyl chloride (282 mg, 0.76 mmol) and TEA (0.46 mL). The resulting solution was stirred at r.t. for 2 days. NaHCO_3_ was added and the mixture was extracted with DCM. The organic portions were washed with 1 M HCl, dried (MgSO_4_), filtered and after evaporation *in vacuo*, the residue purified using flash chromatography (0–10 % MeOH in EtOAc) to afford the title compound as a cream oil (66 mg, 21 %): R_f_ 0.65 (EtOAc); HPLC: *t*_r_=1.65 min (90 % acetonitrile in water), 99 %; ^1^H NMR (CDCl_3_, 400 MHz,): δ (Multiple signals observed due to restricted rotation and therefore the presence of rotamers) 1.62–1.79 (3H, m, CH_2_ and 1/2 CH_2_), 1.98 (1.3H, s, CH_3_), 2.02 (1.7H, s, CH_3_), 2.25–2.32 (1H, m, 1/2 CH_2_), 2.66 (1H, t, *J*=12.0 Hz, 1/2 CH_2_), 2.75–2.84 (1H, m, 1/2 CH_2_), 3.04–3.11 (1H, m, 1/2 CH_2_), 3.23–3.31 (2H, m, CH_2_), 3.67–3.93 (3H, m, CH_2_ and CH), 4.38–4.63 (3H, m, CH_2_ and 1/2 CH_2_), 7.20 (2H, t, *J*=9.6 Hz, ArH), 7.30–7.40 (2H, m, ArH), 7.42–7.51 (3H, m, ArH), 7.60–7.65 (1H, m, ArH), 7.99 (1H, m, ArH); ^13^C NMR (CDCl_3_, 101 MHz): 21.4 (CH_3_), 28.1, 28.3, 28.4, 28.7, 32.5, 34.2 (CH_2_), 38.4, 39.0 (CH), 40.5, 40.8, 42.9, 43.1, 45.4, 45.5, 45.7, 45.8 (CH_2_), 124.2, 124.4 (ArC), 126.0, 126.3, 126.4, 126.7, 128.3, 128.5, 128.7, 128.8, 128.9, 129.0, 129.1, 129.4, 129.6 (ArCH), 135.0, 135.2, 144.7, 146.2, 147.1, 154.8, 156.3, 168.9 (ArC), 172.5, 173.0 (CO); LCMS: *t*_r_=0.93 min (95 % MeOH in water), *m/z* M+H 414.20; HRMS: Calcd for C_27_H_28_N_2_O_2_ (M+H)^+^414.2176, found (M+H)^+^414.2192.

## Supporting Information

Full X‐ray crystallography table of data for **9**.

## Conflict of interest

The authors declare no conflict of interest.

## Supporting information

As a service to our authors and readers, this journal provides supporting information supplied by the authors. Such materials are peer reviewed and may be re‐organized for online delivery, but are not copy‐edited or typeset. Technical support issues arising from supporting information (other than missing files) should be addressed to the authors.

Supplementary
