# Supplementary material for: Rapid and Efficient Microwave‐Assisted Friedländer Quinoline Synthesis
Source: ChemistryOpen. 2020 Nov 5;9(11):1113–22. doi: 10.1002/open.202000247 (PMC7643340; doi:10.1002/open.202000247)
Supplement: Supplementary file 1 — Supplementary [file OPEN-9-1113-s001.pdf]

ChemistryOpen

Supporting Information

Rapid and Efficient Microwave-Assisted Friedländer Quinoline Synthesis

Helen V. Bailey, Mary F. Mahon, Nigel Vicker, and Barry V. L. Potter*

Supplementary Information

Table S1: X-ray crystallography data for 9

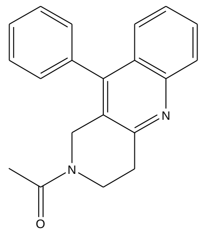

Compound	**9**
Empirical formula	$C_{20}H_{20}N_2O_2$
Formula weight	320.38
Temperature/K	150.15
Crystal system	orthorhombic
Space group	*Pbca*
a/Å	13.3600(2)
b/Å	15.2190(3)
c/Å	15.6200(3)
α/°	90
β/°	90
γ/°	90
Volume/Å^3	3175.95(10)
Z	8
ρ_{calc}g/cm^3	1.340
μ/mm^{-1}	0.087
F(000)	1360.0
Crystal size/mm^3	0.6 × 0.5 × 0.35
Radiation	MoKα (λ = 0.71073)
2θ range for data collection/°	8.028 to 55
Index ranges	-17 ≤ h ≤ 17, -19 ≤ k ≤ 19, -20 ≤ l ≤ 20
Reflections collected	52442
Independent reflections	3638 [R_{int} = 0.0784, R_{sigma} = 0.0435]
Data/restraints/parameters	3638/2/227
Goodness-of-fit on F^2	1.056
Final R indexes [I>=2σ (I)]	R_1 = 0.0430, wR_2 = 0.1003
Final R indexes [all data]	R_1 = 0.0783, wR_2 = 0.1135
Largest diff. peak/hole / e Å^{-3}	0.27/-0.23
